# Dietary intake and nutritional status of patients with phenylketonuria in Taiwan

**DOI:** 10.1038/s41598-020-71361-8

**Published:** 2020-09-03

**Authors:** Hui-Ling Weng, Feng-Jung Yang, Pey-Rong Chen, Wuh-Liang Hwu, Ni-Chung Lee, Yin-Hsiu Chien

**Affiliations:** 1 Department of Dietetics, National Taiwan University Hospital, Taipei, Taiwan; 2 School of Nursing, College of Nursing, National Taipei University of Nursing and Health Sciences, Taipei, Taiwan; 3 Department of Medical Genetics, National Taiwan University Hospital, Taipei, Taiwan; 4 Graduate Institute of Clinical Medicine, College of Medicine, National Taiwan University, Taipei, Taiwan; 5 Rare Diseases Center and Department of Internal Medicine, National Taiwan University Hospital Yun Lin Branch, Douliu, Taiwan; 6 Department of Pediatrics, National Taiwan University Hospital, Taipei, Taiwan

**Keywords:** Clinical genetics, Medical genetics, Endocrinology, Endocrine system and metabolic diseases

## Abstract

Phenylalanine hydroxylase (PAH) deficiency leads to phenylalanine accumulation and results in phenylketonuria (PKU). Phenylketonuria can contribute to severe inability such as mental impairment. Early diagnosis and dietary intervention can have beneficial effects on maintaining normal neural and cognitive function in patients with PKU. However, a long-term low phenylalanine diet may put children at risk of malnutrition. A food supplement was therefore used for children with PKU under dietician supervision according to dietary reference intakes (DRIs). In this cross-sectional study, we enrolled patients with PKU and age-matched controls to compare their anthropometry data [weight, height, body mass index (BMI), and body composition using bioelectrical impedance analysis (BIA)], and correlated it with their dietary intake based on 24-h dietary recall. For continuous parameters, the data were expressed as median ± standard deviation (SD), and the Mann–Whitney *U* test was used to test the difference among the groups. Correlation by natural proteins, body fat, and fat-free mass were evaluated using the Pearson correlation coefficient. Twenty-two participants diagnosed with PKU (ages 8–27 years; mean 15.23 ± 5.23) and a control group of 22 non-PKU participants (ages 8–39 years; mean 19.73 ± 10.6) were recruited for this study. Between the two groups of participants, no significant difference was found in height, weight, BMI, muscle mass, or fat mass. The percentage of natural protein has no effect on body composition. We found a significant positive correlation between the total protein intake percentage of DRIs and muscle mass (r = 0.491, p = 0.020) and a significant negative correlation in the total protein intake percentage of DRIs and fat mass (r = -0.475, p = 0.025) in participants with PKU. There were no significant differences in body composition and nutrition intake between patients with PKU (under metabolic control) and healthy subjects. Thus, giving proper nutrition treatment may have beneficial effects on body growth and nutrition status in patients with PKU in Taiwan.

## Introduction

Phenylketonuria (PKU) refers to an error in phenylalanine (Phe) metabolism. The classical form is characterized by a deficiency in phenylalanine hydroxylase (PAH), a hepatic enzyme responsible for hydroxylation from Phe to tyrosine (Tyr). As a result, excessive Phe accumulates, causing permanent damage to the brain and central nervous system if left untreated^1,2^. Early diagnosis and lifelong nutritional intervention will not only prevent the manifestation of these detrimental effects but also ensure normal cognitive development^3,4^. PKU incidence among Taiwanese population is 1 per 34,000 live births to 1 per 40,000 live births. Since 1986, newborn screening including PKU became mandatory according to the national public health policy. Most of the PKU incidence has since then been diagnosed at the earliest stage after the implementation of newborn screening test^5^. This significantly reduces financial and family burdens national wide as early intervention is done to prevent further complications caused by PKU^6,7^. In Taiwan, patients with PAH deficiency tend to have milder phenotype and therefore high Phe tolerance^8^.

Nutritional therapy for PKU includes a diet containing a Phe-free formula (that includes all other amino acids), nutritional supplement (micronutrients), and low protein-starch foods. This therapy ensures adequate intake of protein, energy, vitamins and mineral among PKU patients^9,10^. As a consequence, growth retardation is prevented when profound intervention is achieved. In many cases, suboptimal PKU dietary management causes many concerns among this specific population. Affected individuals with poor diet management may result in a high Phe plasma level, which interferes with the production of hormone and cytokines such as catecholamines and adiponectin. Contrarily, individuals with extremely strict Phe diets are often found not to have sufficient intake in essential amino acids and other micronutrients. A strict Phe diet is not the problem, but not taking an amino acid mixture is the main problem. Net protein utilization is compromised as the result of l-amino acids and mixture protein deficiency^3,10–12^.

In Taiwan, prescribed nutritional supplements for PKU , such as a Phe-free formula and l-amino acid, are fully supported by the government. Not only that, low-protein medical foods such as low-protein rice and low-protein noodles are partially supported as well. Low protein medical foods are essential starch substitutes in patients’ daily meals particularly for children with high demand in energy and nutrient intake. Additionally, many common Taiwanese low protein-starch foods such as vermicelli, sago, grass jelly etc. are often incorporated in low Phe diet to fulfill satiety as well as energy intake.

Some of the previous researches stated that affected individual undertaking low-phenylalanine diet showed growth retardation^13,14^. Other literature showed a correlation between low Phe diet adherence with overweight^1,5,7^ or obesity. Recent studies even pointed out that there was no significant difference in growth and body composition between affected and normal individual^15–18^. These contradicted findings pointed out that in fact, low Phe diet is not the only factor determining child growth. Many perspectives of intervention also contribute to child growth for example, overall nutritional intake, disease management and physical activity level^4,19–21^.

As patients get older, it is harder to adhere to a strict low Phe diet—one of the reasons being wider availability of food choice^17,18^. Therefore, adherence to lifelong low Phe diet is very challenging for the affected individuals and also the whole medical team as well. In Taiwan, such a sound medical support environment, it is worthwhile to explore the health outcome and nutritional status of PKU patients. Bioelectrical impedance assessment (BIA) is an efficient method to provide accurate measurement of body composition such as fat mass and muscle mass^17,18^. This method is applicable for people age from 6 years old onwards^22–25^. Although many types of research have been done to examine the nutritional status of children with PKU in many countries, there is a lack of study conducted among Taiwanese group in specific. Significant dietary and cultural differences are taken into consideration in this study to provide a valuable reference for clinical dietary therapy and national public health organizations in Taiwan.

## Method

This cross-sectional study was approved by the Institutional Review Board of National Taiwan University Hospital. A total of forty-four participants were enrolled through National Taiwan University Children’s Hospital. All were identified through newborn screening. Twenty-two participants were diagnosed with PKU (ages 8–27 years; 10 males, 12 females; mean 15.23 ± 5.23, Table 1), in which 13 of these diagnosed patients (55%) received a Phe-free formula supplement. The other 22 participants with same age group and gender proportion were enrolled as a control group (8–39 years old; 10 males, 12 females; mean 19.73 ± 10.6). All dietary rercommandations were base on Dietary Reference Intakes (DRIs), set of reference values used to plan and assess nutrient intakes of healthy people and in designing and evaluating research studies and results^26^. All research procedures followed the directives of the Declaration of Helsinki.

**Table 1:** Subject characteristics, growth, and body composition.

Item	PKU patients (N = 22)	Healthy controls (N = 22)	*p*-value
**Age years ± SD**	15.23 ± 5.23	19.73 ± 10.6	0.3392
**Sex**			
Male	10 (45%)	10 (45%)	1.00
Female	12 (55%)	12 (55%)	
**Growth**			
Height z-scores	− 0.045 ± 0.096	− 0.041 ± 0.08	0.9719
Weight z-scores	− 0.611 ± 1.381	− 0.871 ± 1.279	0.4047
BMI z-scores	− 0.639 ± 1.297	− 0.639 ± 1.19	0.4248
**Body composition**			
Muscle mass, %	73.391 ± 8.789	75.512 ± 7.421	0.4455
Fat mass, %	20.741 ± 8.900	18.673 ± 7.529	0.4635
**Dietary inake**			
Natural protein, g/kg/day	0.874 ± 0.602		
Total protein, g/kg/day	1.265 ± 0.592		
Energy intake, kcal/kg/day	41.909 ± 15.075		
Protein intake of DRIs%	105.448 ± 33.41		
Energy intake of DRIs%	103.514 ± 22.087		

^a^*p*-values were calculated using the Mann–Whitney *U* test, Fisher’s exact test or Chi-square test.

^b^Z-scores = (raw score-the mean of the controls)/the standard deviation of the controls.

*p value < 0.05.

Height, weight, BMI and BIA measurements were collected from all of the participants by dieticians. In addition, 24-h food recall was conducted for food analysis.

### Statistical analysis

Statistical analyses were performed with SAS Version 9.4 (SAS) and Prism Version 8.3.1(322) (GraphPad). All data collected were presented as mean with standard deviation. For continuous variables, Mann–Whitney *U* test was used for analyzing differences between independent groups. Fisher’s exact test or Chi-square test was used to find the differences in the distribution of each group. The associations between natural proteins, body fat and fat-free mass were evaluated with Pearson correlation coefficient. Moreover, the level of significance was set at p < 0.05.

### Ethics approval and consent to participate

The study was approved by NTUH’s institutional ethical committee (NTUH IRB-201408018RINA) and informed consent was acquired from all participants.

### Consent for publication

All authors consent to the publication of this final version of the manuscript.

## Results

### Energy and protein intakes

PKU patients have similar growth development compared to healthy individuals during follow-up. Table 1 shows that all of the PKU patients had total protein of 105.4 ± 33.5% of dietary reference intake (DRIs) and total energy of 103.5 ± 22.1% of DRI. These values are above DRIs and at the same time higher than the recommended safe intake according to many health organizations (Food and Agriculture Organization of the United Nations, World Health Organization, United Nations University). Additionally, 71% (70.90 ± 31.24) of protein consumed by PKU patients was from medical food and 29% from natural food.

Table 2 showed a comparison of protein intakes between patients consuming a Phe-free formula and patients who did not consume a Phe-free formula. As predicted, patients consuming a Phe-free formula had a higher total protein intake (1.29 ± 0.54 vs. 1.23 ± 0.68) and lower natural protein intake (0.57 ± 0.32 vs. 1.23 ± 0.68).

**Table 2:** The comparisons between dietary intake and growth and body composition in PKU patients.

Subject characteristics	PKU patients		*p*-value
	Phe-free formula (N = 12)	Non-Phe-free formula (N = 10)	
**Age years ± SD**	15.75 ± 5.92	14.60 ± 4.48	0.69
**Growth**			
Height z score^b^	− 0.839 ± 2.201	− 0.694 ± 1.755	0.7713
Weight z score^b^	− 0.623 ± 1.403	− 0.597 ± 1.426	0.9474
BMI z score^b^	− 0.318 ± 1.511	− 0.43 ± 1.062	0.4097
**Body composition**			
Muscle mass (%)	73.15 ± 8.5	73.68 ± 9.5579	0.9474
Fat mass (%)	20.975 ± 8.603	20.46 ± 9.705	0.9229
**Dietary intake**			
Natural protein, g/kg/day	0.574 ± 0.316	1.234 ± 0.679	0.004
Total protein, g/kg/day	1.292 ± 0.54	1.233 ± 0.678	0.7223
Energy intake, g/kg/day	42.279 ± 16.159	41.466 ± 14.518	0.9742
Protein intake of DRIs (%)	109.672 ± 28.606	100.379 ± 39.395	0.5824
Energy intake of DRIs (%)	106.142 ± 21.289	100.361 ± 23.75	0.5387

^a^*p*-values were calculated using Mann–Whitney *U* test.

^b^Z-scores = (raw score-the mean of the controls)/the standard deviation of the controls.

### Anthropometry and body composition analysis

Table 1 shows no significant differences in age and gender between PKU patients and the control group. It also showed no significant differences in height (z score, − 0.10 ± 1.14 vs. 0.00 ± 0.10, *p* = 0.92), weight (z score, 0.11 ± 0.94 vs. 0.00 ± 1.00, *p* = 0.5), and BMI (z score, 0.30 ± 1.07 vs. 0.00 ± 1.00, *p* = 0.29). In body composition data, no significant difference was found in muscle mass (73.39 ± 8.79 vs. 75.51 ± 7.42, *p* = 0.37) and fat mass (20.74 ± 8.90 vs. 18.67 ± 7.53, *p* = 0.45).

### Association between dietary intake, anthropometry data and body composition

Even though a significant difference was found in natural protein intake between the two groups (Phe-free formula and non-Phe-free formula) as shown in Table 1, both anthropometry data (weight, height, and BMI) and body composition (muscle mass and fat mass) showed no significant difference between these groups.

**Figure 1:**
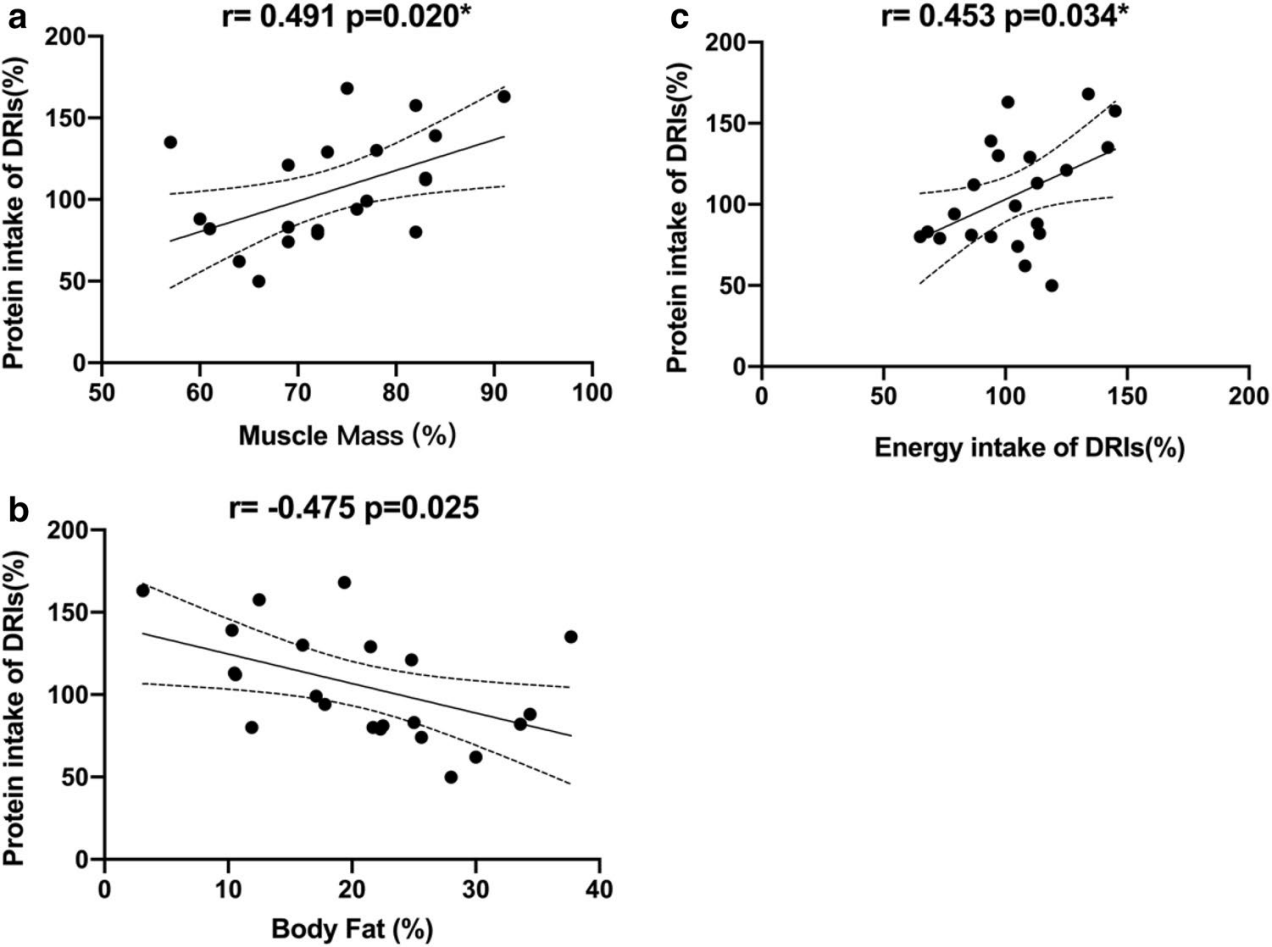
The correlation between protein intake and body composition in PKU patients. *DRIs* dietary reference intakes.

Furthermore, positive correlation was found between total protein intake percentage of DRIs and muscle mass (r = 0.491, p = 0.020, Fig. 1a) and significant negative correlation in total protein intake percentage of DRIs and fat mass (r = − 0.475, p = 0.025*, Fig. 1b). Besides, total protein intake percentage of DRIs was correlated positively with energy intake of DRIs (r = 0.453 p = 0.034*, Fig. 1c). Natural protein percentage has no correrlation to muscle mass (r = − 0.007, p = 0.974) and fat mass (r = 0.009, p = 0.969). Total protein intake percentage of DRIs is most important factor of PKU diet for body composition.

Our results showed that PKU patients in Taiwan had reached to protein and energy DRIs while consuming Phe restricted diets as mentioned above. Overall anthropometry data and body composition data were no different compared to the control group thus malnutrition did not exist in patients diagnosed with PKU.

## Discussion

Many studies conducted in the years between 1980 and1990 had a common finding of linear developmental impairment among PKU patients. The contrary finding was shown in a study done in 2010 as the growth development was found to be no difference between low Phe diet group and healthy individuals^15,16,27,28^. This is reflective to our finding as the height z-score was not statistically different between PKU patients and the control group regardless of height z score being − 0.10 ± 1.14 in PKU patients. Furthermore, when using the overweight scale from the US Centers for Disease Control and Prevention (CDC)^29^ as a benchmark, no sign of obesity was observed in the PKU group. To extend our research, comprehensive body composition data were obtained via BIA. In the PKU group, the fat mass percentage was higher than in the control group without significant difference. Additional evidence supports our finding that PKU does not interfere with growth development nor contribute to obesity^30–34^. In Taiwan, patients with PKU are advised to have plasma Phe levels less than 360 μM before age 3 years, with slight increases up to 600 μM if it is not feasible to keep below 360 μM. All patients maintained good blood levels initially, but the levels increase with age^35^. Instead of the average Phe level, we collected only the current Phe level in this study as a reference.

A study done by Huemer et al. has found a positive correlation between muscle mass and natural protein intake among PKU patients^15^. It stated that total protein intake greater than 1.5–2.6 g/kg is beneficial for body development. However, when total protein intake is greater than 2.6–3.5 g/kg, no benefit is shown. This is similar to our finding as the results above have shown a positive correlation between natural protein intake and muscle mass while negative correlation is shown between natural protein intake and fat mass. The main sources of protein in PKU nutritional therapy in Taiwan include natural food sources and Phe-free formula. Previous research stated concern of possible developmental retardation among patients consuming a Phe-free formula as the formula is less absorptive to the digestive system. Evans et al. suggested that PKU patients should maximize natural protein intake to achieve health body composition^36,37^. One of the mechanisms behind this theory is that the satiety will be increased with maximized natural protein intake hence reduced chance of consuming excessive energy intake. Secondly, increased dietary protein-induced thermogenesis helps with Resting Energy Expenditure. The other mechanism for maximal natural protein intake is the increased production of growth hormone and IGF 1 which lead to increased muscle mass growth. Lastly, maximal intake will stimulate muscle protein synthesis to maintain lean muscle mass^36,37^.

Controversial findings still exist regarding the correlations between PKU nutritional therapy, growth retardation, and body composition. Despite these controversies, it is recommended by the experts that total protein intake and energy intake should be higher than the recommended dietary allowance in pediatric low Phe dietary therapy^38^. On average, the enrolled PKU group consumed 1.3 g/kg/day of total protein (110% of DRIs) which meets the recommendation by experts. Given that no signs of malnutrition and growth retardation were found in the existing PKU patients, we are confident that the PKU patients will adhere to the current diet plan and be advised to meet dietary reference intake of protein.

## Conclusion

There were no significant differences in body composition and nutrition intake between patients with PKU (under metabolic control) and healthy subjects. Thus, giving proper nutrition treatment may have beneficial effects on body growth and nutrition status in patients with PKU in Taiwan.

### Limitation

This is a retrospective cohort study to review the quality of care among patients with PKU at a teaching hospital in Taiwan. The sample size is relatively small. A larger sample size and collabration with more hospitals would enable a more thorough examination of the dietary needs of PKU patients.

## Data Availability

Original data is available upon request.

## Abbreviations

PKU: Phenylketonuria
PAH: Phenylalanine hydroxylase
Phe: Phenylalanine
Tyr: Tyrosine
BIA: Bioelectrical impedance assessment
BMI: Body mass index
BW: Body weight
BH: Body height
DRIs: Dietary reference intakes
CDC: Centers for Disease Control and Prevention
